# Levels of serum B12, folic acid and homocysteine in thromboembolic diseases on admission to the Emergency Department

**DOI:** 10.1186/cc9434

**Published:** 2011-03-11

**Authors:** A Bayır, K Uçar Karabulut, A Ak

**Affiliations:** 1 Selçuk University, Selçuklu Faculty of Medicine, Emergency Department, Konya, Turkey; 2 Şırnak State Hospital, Şırnak, Turkey

## Introduction

The aim of this study was to compare with control and each other the levels of serum B12, folic acid and homocysteine at admission in the cases with thromboembolic diseases.

## Methods

This study included 100 subjects with acute myocardial infarctus (AMI), acute pulmonary embolism, deep vein thrombosis, ischemic cerebrovascular disease (ICD), acute mesentery embolism, and peripheric artery embolism (PAE), and 110 healthy voluntary subjects were included in the control group. Vitamin B12, folic acid and homocysteine levels were examined in the blood samples obtained at admission, The data were loaded onto SPSS 16 for Windows program. *P *≤ 0.05 was considered significant.

## Results

Mean serum homocysteine and plasma vitamin B12 levels were significantly higher in the patient group than the control group (*P *= 0.002 and 0.000 respectively). There was no significant difference in the levels of folic acid between the patient and control groups. Mean serum B12 values of the AMI and ICD groups in the patient group were significantly lower than those of the control group (*P *< 0.05). Serum folic acid values of the PAE and AMI groups were considerably lower than the control group (*P *< 0.05). Plasma homocysteine levels were significantly higher in all patient groups according to their diagnosis than the control group (*P *< 0.05).

## Conclusions

Mean serum homocysteine and plasma vitamin B12 levels were significantly higher in the patient group than the control group (*P *= 0.002 and 0.000 respectively). There was no significant difference in the levels of folic acid between the patient and control groups. Mean serum B12 values of the AMI and ICD groups in the patient group were significantly lower than those of the control group (*P *< 0.05). Serum folic acid values of the PAE and AMI groups were considerably lower than the control group (*P *< 0.05). Plasma homocysteine levels were significantly higher in all patient groups according to their diagnosis than the control group (*P *< 0.05).
